# Surveillance of Injury Types, Locations, and Intensities in Male and Female Tennis Players: A Content Analysis of Online Newspaper Reports

**DOI:** 10.3390/ijerph182312686

**Published:** 2021-12-01

**Authors:** Rabiu Muazu Musa, Isyaku Hassan, Mohamad Razali Abdullah, Mohd Nazri Latiff Azmi, Anwar P. P. Abdul Majeed, Noor Azuan Abu Osman

**Affiliations:** 1 Centre for Fundamental and Continuing Education, Universiti Malaysia Terengganu, Kuala Nerus 21030, Malaysia; rabiu.muazu@umt.edu.my; 2 Faculty of Languages and Communication, Gong Badak Campus, Universiti Sultan Zainal Abidin, Kuala Nerus 21300, Malaysia; mnazrix@gmail.com; 3 East Coast Environmental Research Institute, Gong Badak Campus, Universiti Sultan Zainal Abidin, Kuala Nerus 21300, Malaysia; razaliabdullah@unisza.edu.my; 4 Innovative Manufacturing, Mechatronics and Sports Laboratory, Faculty of Manufacturing and Mechatronic Engineering Technology, Universiti Malaysia Pahang, Pekan 26600, Malaysia; amajeed@ump.edu.my; 5 Department of Biomedical Engineering, Faculty of Engineering, University of Malaya, Kuala Lumpur 50603, Malaysia

**Keywords:** injury surveillance, tennis sports, chi-square test, media content analysis, newspapers, injury prevention, tennis-related exercise, media analysis of injury, tennis injury in male and female players

## Abstract

The popularity of modern tennis has contributed to the increasing number of participants at both recreational and competitive levels. The influx of numerous tennis participants has resulted in a wave of injury occurrences of different types and magnitudes across both male and female players. Since tennis injury harms both players’ economic and career development, a better understanding of its epidemiology could potentially curtail its prevalence and occurrences. We used online-based tennis-related injury reports to study the prevalence, location types, and injury intensities in both male and female tennis players for the past five years. It is demonstrated from the chi-square analysis that injury occurrences are significantly associated with a specific gender (χ2(18) = 50.773; *p* = 0.001), with male players having a higher risk of injury manifestation (68.10%) as compared with female players (31.90%). Nonetheless, knee, hip, ankle, and shoulder injuries are highly prevalent in both male and female players. Moreover, the injury intensities are distributed across gender (χ2(2) = 0.398; *p* = 0.820), with major injuries being dominant, followed by minor injuries, whilst a few cases of career-threatening injuries were also reported. It was similarly observed that male players recorded a higher degree of both major, minor, and career-threatening injuries than female players. In addition, male players sustained more elbow, hip, knee, shoulder, and thigh injuries than female players. Whereas, female players mostly suffered from Achilles and back injuries, ankle and hamstring injuries affected both genders. The usage of online newspaper reports is pivotal in characterizing the epidemiology of tennis-related injuries based on locations and gender to better understand the pattern and localization of injuries, which could be used to address the problem of modern tennis-related injuries.

## 1. Introduction

A recent position statement on burnout and overuse injuries in the sporting context indicates that elevated exercise volumes, overscheduling, and adolescent development spurts are contributing factors for the escalation of overuse injuries in sports [1,2,3]. Hitherto, recent tennis data highlighted an alarming escalation in the rate of injuries reported during competitions, which calls for an immediate intervention [4,5]. Research indicates the need for systematic studies to better understand tennis-related injuries and to design valuable strategies for preventing the occurrence of such injuries [4,6,7]. However, the collection of accurate data for tennis injury analysis remains a challenge [8]. Additionally, robust methods of injury data collection are required to allow for sophisticated analysis of tennis-related injuries [9]. In this regard, research has shown the importance of mainstream media, particularly newspapers, in providing useful data for injury analysis [10,11]. It has also been reported that newspapers cover more injury-related events than other sources [11]. When an injury occurs, such an incident is likely to become a newsworthy event or topic of interest amongst audiences. It is worth highlighting that newspapers are shown to be reliable and strong sources of information [12,13]. The information obtained from newspapers is considered vital in providing a variety of data to both researchers and practitioners to aid decision-making [14]. As such, newspapers could serve as a potential source of injury data [15,16].

The popularity of modern tennis has become global, with approximately 1.7% of the world population playing the sport, and well over 200 countries are affiliated with the International Tennis Federation [17]. This global popularity of tennis has witnessed a steady increase in the number of players, with both males and females being registered or licensed [18]. In contrast with many other sports, the length of actual play during a tennis match is not limited by time. As a result, matches can last for several hours, necessitating hundreds of short as well as explosive bursts of energy [19]. The recurrent demands for both aerobic and anaerobic energy, combined with various strokes, result in the occurrence of a variety of injury profiles [20]. These injuries do not only cause interruptions to the training and tournament fixtures but also inflict psychological stress and an economic condition that consequently harm the players’ career development [7,21].

Although previous studies have analyzed tennis-related injuries using different designs [6,8,22], the authors acknowledge certain challenges in the accumulation of readily available and relevant injury data that could be applied for analysis to aid decision-making. Hitherto, none of these studies focused on a media-based analysis of tennis-related injuries. Furthermore, a significant scarcity of injury-related data has been reported in developing countries [11,23]. Therefore, via media content analysis, this study examines tennis-related injuries obtained from selected Nigeria-based online newspapers from January 2015 to December 2020. The newspapers were selected based on their online readership and popularity. To the best of our knowledge, this study is an initial attempt to investigate injury prevalence in tennis from the mainstream media perspective. Specifically, this study aimed to examine injury occurrences and exposure to tennis-associated injuries based on gender, type, location, and intensity. It is envisaged that the surveillance of nature, types, and localization of tennis-related injuries could aid medical teams and health personnel to set-up a preventive measure geared towards reducing the prevalence of tennis injuries for both male and female players.

## 2. Materials and Methods

A detailed explanation of the methodological approach adopted in the current study is segmented under the following sub-headings:

### 2.1. Content Analysis Approach

This study employed a content analysis approach to look at tennis-related injuries in five major Nigerian online newspapers. Content analysis was selected due to its capacity to investigate various phenomena via media content. This is because content analysis contains specific media content that can be processed to generate valuable data. This approach also considers text-based correspondence and allows for quantitative and qualitative analyses [24,25]. Content analysis is recognized as a research method based on facts, as opposed to other methods such as discourse analysis [26]. A “content analysis takes texts and analyses, reduces and interrogates them into a summary form through the use of both pre-existing categories and emergent themes to generate or test a theory” [27].

### 2.2. Sampling Method and Years of Coverage

Five national newspapers with the highest readership and online popularity were selected for analysis: *Vanguard*, *Punch*, *The Nation*, *The Sun*, and *This Day*. These newspapers are the top daily English language publications in Nigeria (Top Ten Nigerian Newspapers, 2019). Unlike specialized international sports media that mostly focus on results and tournaments, newspapers tend to cover tennis-related stories locally, nationally, and internationally [28,29]. Thus, rich and large-scale tennis injury data could be gathered from online newspapers. The Nigerian newspapers were considered in this study due to their presence on both print and digital platforms. Their availability in these versions enables them to reach a global audience covering local, international, and regional sports news [30].

News articles focusing on tennis-related inquiries were collected from the respective digital archives of the selected newspapers between January 2015 and September 2020. This time represents a period with various tennis tournaments across the globe, such as Rio Tennis Olympics, Wimbledon Championships, Australian Open, and the US Open. The time frame was chosen to make the data into a manageable size for analysis.

### 2.3. Parameters Search

The news articles were collected using “tennis” and “injury” as keywords. All news articles identified using this method were included in the content analysis. Only straight news and feature stories were selected for analysis. A total of 113 relevant news articles were generated. Online news articles were considered in this study as readers can attend to online news at any time [31,32]. Additionally, newspapers are recognized as a more detailed and reliable source of data than traditional datasets [11,15]. Although data gathered from news content are considered qualitative, they can be computed to obtain quantifiable results. Thus, content analysis is mainly characterized as a qualitative versus quantitative research method [24]. Moreover, it has been reported that news content can provide valuable information on the prevalence and dimensions of unintentional injuries [15].

### 2.4. Coding Technique and Reliability Analysis

At the initial stage, each article in the original sample was read for relevance based on headlines and news content. The relevant content of each news article was studied and coded using a hierarchical code system. The hierarchical code system is advantageous as it represents a huge amount of information in a few digits [24]. Subsequently, an Excel database was generated with four categorical variables: injury locations, types, and intensities, as well as gender. Information related to players’ age was also collected. The average age of players involved in the current study was 26 years. To ensure consistency, inter-coder reliability was employed. Two independent coders who were anonymous to each other and had no prior knowledge of the intention of the study were involved in assessing the reliability of the data gathered. Since two raters were involved in the analysis, the intercoder agreement was determined using Cohen’s kappa analysis, as suggested in previous research [33]. The inter-coder reliability outcomes revealed a Cohen’s kappa agreement of 1.000 concerning players’ gender as well as 0.961 for injury types and 0.840 for injury intensities. Cohen’s kappa has been considered as a robust method of reliability measurement for content analysis [34,35,36].

Moreover, the news articles were cross-checked to avoid double entry of data. In addition, the emerging themes were verified to avoid reproducing media content or revealing dominant themes containing certain tennis-related information. Specifically, the researchers aimed to explore tennis-related injuries concerning injury types, locations, and intensities, as well as players’ gender throughout the coding process. This method involved subjective judgment, but inter-coder reliability helped to achieve a systematic analysis (Supplementary Materials) [37].

### 2.5. Employment of Chi-Square Analysis in the Study

The chi-square test was essentially developed to facilitate the analysis of categorical data. In other words, the chi-square test caters to a dataset that is counted and categorized. Thus, the test does not work with parametric or continuous numeric data. It is worth highlighting that the data set required to run the chi-square test should not be in the form of percentages, percentiles, or relative frequencies but rather in the form of frequency, i.e., count datasets [38]. In the current investigation, chi-square analysis was employed to study the incidence, exposure, and status of tennis-associated injuries as covered by the selected online newspapers. Essentially, the test is applied to examine tennis-related injury occurrences based on gender (male vs. female) and injury types as well as injury intensities (major, minor, career-threatening). The chi-square analysis was performed using the following equation:

(1) $$\chi^{2}=\overset{n}{\underset{i=1}{\sum }}\frac{{\left(O_{i}-E_{i}\right)}^{2}}{E_{i}}$$

where *O* refers to the observed frequency, whilst *E* reflects the expected frequency. The expected count is subtracted from the observed counts to ascertain the difference between the two (residual). The square of the number is then computed to eliminate the positive and negative values. The result is then divided by the expected frequency to normalize the larger and smaller counts such that the chi-square value does not unnecessarily increase due to the existence of a larger dataset. The Σ implies the summation of every *i* sample calculated for each cell within the table. Where the expected count in the column is less than 5 percent, a likelihood ratio is reported [39]. All the inferences in the present study were drawn at an alpha (α) level of ≤0.05 using the SPSS statistical software package (SPSS Inc., Chicago, IL, USA).

## 3. Results

Table 1 projects the distribution of injury types in association with the players’ gender. It can be observed that injury occurrences were significantly associated with male players. As such, male tennis players tended to be exposed to a higher risk of varying injuries when compared with female players (*p* < 0.001). Similarly, it can be seen that knee, hip, ankle, back, and shoulder injuries were found to be highly prevalent in both male and female players.

**Figure 1:**
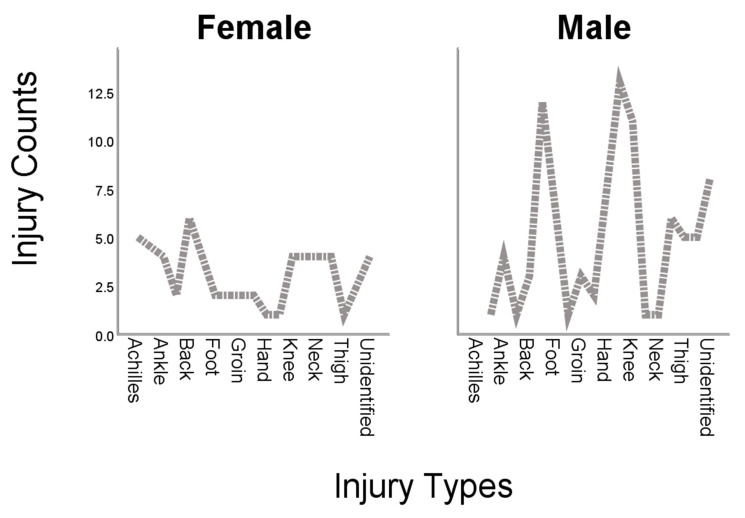
Injury types and their prevalence among male and female tennis players.

Figure 1 displays a graphical representation of the injury types and their prevalence amongst male and female players. It could be observed from the figure that certain types of injuries such as elbow, hip, knee, shoulder, and thigh are especially common in male players, while Achilles and back injuries are peculiar to female players. However, ankle, as well as hamstring, were found or reported in both genders.

Table 2 tabulates the incidence of injury intensities based on the players’ gender. It can be seen that the injury status is distributed with regard to both males and females (*p* > 0.05). Nonetheless, larger proportions of injury intensities across the gender are major injuries (93), while minor injuries are relatively high (13). Regrettably, some cases of career-threatening injuries were observed (7) with regard to gender.

## 4. Discussion

The present study sought to investigate the occurrences and exposure of tennis-related injuries depending on the types, intensities, and locations in both male and female tennis players. Owing to the capacity of online-based newspaper content in offering timely and easily accessible injury-related news to quantify and characterize multiple incidents, a media-based content analysis was used in the current investigation to address the issue of data paucity.

The findings of the current investigation revealed that male players possess a higher risk of getting injured (68.10%) as compared with female players (31.90%). This finding conforms with the findings reported by preceding researchers, where male tennis players were found to be more frequently injured as compared with female players [18,40,41]. This outcome might be due to the intensity applied and the playing styles adopted by different genders. For instance, female players were reported to exhibit a playing style that emphasizes a more defensive backline as opposed to male players [41]. It has also been speculated that differences in the movement patterns that exist between males and females could explain the variation in the injury occurrences. The male athletes tend to apply both feet during a change of direction, whilst female athletes tend to employ one foot when executing a sudden change of direction [42,43]. Similarly, during the landing process, female athletes are inclined to land in an upright manner, with the knees near to each other [44].

The current study also demonstrated that specific injury types such as elbow, hip, knee, shoulder, and thigh injuries, are more prevalent in male players, while Achilles and back injuries are more common in female players. This is not a coincidence, as it is evident that male tennis players often apply a more direct and aggressive playing strategy, which necessitates the usage of both upper and lower extremities to be highly engaged [45,46]. Consequently, this pattern of play could increase the risk of injuries due to the high-velocity movements and the repetitive arm actions required during the execution of tennis-related skills [47,48]. Hence, these injuries are found to be higher in males owing to the overuse as well as rapid and sudden recruitment of body parts [49]. It is, therefore, no surprise that male players were found to be associated with a high degree of major and minor injuries. On the other hand, some factors, including anatomical orientation and neuromuscular activity, differ between males and females [50,51]. This variation, when associated with the various demands that different sports impose on male and female athletes, can explain why the risk of certain injuries is higher in male as opposed to female players [52]. It is important to highlight that research targeting the variation in injury risks and occurrences in specific locations in male and female athletes is still in its infancy, and thus more studies are required to elucidate the various types of injuries and their localization in male and female players with respect to sporting activity.

In another perspective, knee, hip, ankle, back, and shoulder injuries were found to be highly prevalent in both male and female players. It could then be deduced that the most prevalent tennis-associated injuries occur in the lower, trunk, and upper parts of the body, respectively. The tennis game could be played on several types of surfaces that constitute clay and grass, as well as hard courts. The nature of the playing surface may predispose the players to certain types of injuries. For instance, it was observed that the ball travels faster on hard courts, and hence the speed of the ball could induce more force to the upper body [20]. There is inadequate research associating the playing surface with frequency or types of injuries. Nonetheless, it was documented that muscle is sensitive to surface rigidity, and thus regular play on a variety of surfaces could be linked to the occurrences of injuries within the lower extremity region [53,54]. Similarly, it was reported that the incidence of knee injury in tennis is considerably high, as the location is subjected to repetitive unusual overload [55]. Unlike other sports such as soccer, tennis involves running and skating, where loading mostly affects the lower extremities [56]. Additionally, tennis does not have a specific predetermined duration, and therefore matches often last several hours with many short bursts of energy [19,20]. This unique profile of tennis stimulates the prevalence of injuries that are considered to be higher within the lower body and trunk as well as upper body as opposed to other non-contact sports [57].

Finally, it is interesting to note that in the current analysis, the chosen online newspapers published more global news on tennis injuries than regional, local, or national news. These results show the perceived significance of tennis-related injuries in the newspapers studied. This outcome is due to the premise of the salience-based technique, which holds that the extent of press attention is critical in determining the degree of importance accorded to the topic being covered [58,59]. Considering this assumption, tennis-related injuries receive tremendous media attention in Nigeria. However, the selected newspapers tend to rely on news agencies, such as Agence France-Presse (AFP), News Agency of Nigeria (NAN), and Reuters, as well as other conventional media including British Broadcasting Corporation (BBC) and Cable News Network (CNN), for information on tennis-related injuries. Therefore, the usage of online newspaper reports is pivotal in characterizing the epidemiology of tennis-related injuries based on locations and gender to better understand the injury pattern and localization that could be used to mitigate the lingering problem of modern tennis-related injuries.

## 5. Conclusions

In the current study, knee, hip, ankle, back, and shoulder injuries are identified as highly prevalent in both male and female tennis players. Understanding the mechanisms through which tennis players encounter injury-related risks, strength and conditioning experts could introduce a variety of exercise programs that may offset the steps toward injury occurrences. As a practical application, therefore, specific injury-prevention exercises such as vastus medialis oblique (VMO) muscle, band work exercise, dynamic and controlled lunges, and one-legged squats could help to strengthen the knee. In addition, clamshells and reversed clamshells, hip abduction/adduction, plyometric lateral stepovers, and elastic tubing kicks could help to prevent hip injuries. Towel curl, toe pull, standing heel raise, and golf ball roll could assist athletes in addressing ankle injuries. Moreover, sit-ups on a stability ball, Russian twist, prone extension and plank, quadruped pointer, and dead bug could help to address back injuries. Additionally, Jobe rotator cuff exercises, side-lying external rotation, prone extension, prone horizontal abduction, prone external rotation, shoulder external rotation (neutral tubing), Shoulder external rotation 90, abduction (tubing), and shoulder 90/90 prone plyometrics exercises could be beneficial to the shoulders [60]. These sets of exercises could be valuable to coaches, athletes, and medical staff in addressing recurrent injuries in the identified regions through targeted training and exercise programs that can prolong and safeguard the players’ careers as well as improve their performance.

### Strength and Limitation of the Study

The detection of the most often injured anatomical regions in tennis is a significant indicator of locations that should be targeted for preventative training by strength and conditioning specialists. In the present study, we determined the likelihood of injury occurrences, the common locations of injury occurrences, and the most highly prevalent injuries as well as the gender that is more prone to injury risks. We suggested some specific injury prevention exercises for the highly prevalent injury sites. This set of exercises could assist coaches and strength and conditioning professionals, as well as athletic trainers in designing suitable programs that could address the injury occurrences and diminish the risks of injuries in tennis players both during training and competition. However, some limitations of the study are acknowledged. We were unable to study the injury occurrences with respect to players’ age, tournament types, and players’ expertise levels. It is believed that the aforementioned variables could provide a more comprehensive analysis of injury-related incidence in tennis. Moreover, there is a need to examine the effect of specific training programs on injury prevention in this type of sport. However, this examination is beyond the scope of the current investigation. It is, therefore, recommended that future research should consider the above variables for injury analysis in tennis players.

## Figures and Tables

**Table 1:** Association of injury types with gender.

Injury Types	Gender		Total
	Male F (%)	Female F (%)	
Achilles	0 (00.00)	5 (100.00)	5
Abdominal	1 (100.00)	0 (0.00)	1
Ankle	4 (50.00)	4 (50.00)	8
Arm	1 (33.30)	2 (66.60)	3
Back	3 (33.30)	6 (66.60)	9
Elbow	12 (100.00)	0 (0.00)	12
Foot	0 (0.00)	2 (100.00)	2
Glute	1 (100.00)	0 (0.00)	1
Groin	3 (100.00)	0 (0.00)	3
Hamstring	2 (50.00)	2 (50.00)	4
Hand	0 (0.00)	1 (100.00)	1
Hip	13 (92.90)	1 (7.10)	14
Knee	11 (73.30)	4 (26.70)	15
Leg	1 (100.00)	0 (0.00)	1
Neck	1 (100.00)	0 (0.00)	1
Shoulder	6 (60.00)	4 (40.00)	10
Thigh	5 (83.30)	1 (16.70)	6
Wrist	5 (100.00)	0 (0.00)	5
Unidentified	8 (66.70)	4 (33.30)	12
Overall Total	68.10%	31.90%	113

χ2(18) = 50.773; *p* = 0.001.

**Table 2:** The incidence of injury intensities across male and female tennis players.

Injury Intensity	Gender		Total
	Male F (%)	Female F (%)	
Major	64 (68.80)	29 (31.20)	93
Minor	9 (69.20)	4 (30.80)	13
Career Threatening	4 (57.10)	3 (42.90)	7
Overall Total (%)	68.10%	31.90%	113

χ2(2) = 0.398; *p* = 0.820.

## Data Availability

The data used are available within the manuscript.

## Supplementary Materials

The following are available online at https://www.mdpi.com/article/10.3390/ijerph182312686/s1.
