# Supplementary material for: Surveillance of Injury Types, Locations, and Intensities in Male and Female Tennis Players: A Content Analysis of Online Newspaper Reports
Source: Int J Environ Res Public Health. 2021 Dec 1;18(23):12686. doi: 10.3390/ijerph182312686 (PMC8656957; doi:10.3390/ijerph182312686)
Supplement: Supplementary file 1 [file ijerph-18-12686-s001.zip › ijerph-1408419-supplementary.pdf]

SN	Types	Position	Context	Gender	Age	Intensity	Responses from organizers	News Sources	Tournament	Coverage
1	1	15	3	2	28	1	2	2	27	1
2	10	16	3	2	28	1	2	2	27	1
3	13	5	3	1	28	1	2	2	27	1
4	7	7	2	2	28	1	2	1	1	1
5	13	6	1	2	28	1	2	1	7	1
6	16	19	3	2	9	2	1	2	6	1
7	13	5	1	1	28	1	2	1	12	1
8	19	19	1	2	1	1	1	1	8	1
9	9	19	1	1	8	1	2	3	11	1
10	3	2	1	2	3	1	2	2	27	1
11	16	19	1	1	8	1	1	2	27	1
12	3	2	3	2	28	1	2	2	13	1
13	11	19	3	2	17	1	2	2	13	1
14	19	19	1	2	28	1	2	2	15	1
15	13	6	1	2	3	1	2	2	17	1
16	12	19	1	2	28	1	1	6	23	1
17	3	19	1	1	8	1	2	6	4	1
18	17	19	3	1	8	2	2	2	8	1
19	5	19	3	2	13	1	2	6	4	1
20	10	16	1	1	28	1	2	2	27	1
21	13	5	1	1	28	1	2	1	12	1
22	19	19	1	2	1	1	2	1	1	1
23	13	5	3	1	10	1	2	2	3	1
24	8	19	1	1	18	1	1	2	27	1
25	5	17	2	2	13	1	2	2	31	1
26	6	19	1	1	28	1	2	2	33	1
27	1	19	1	2	1	1	2	2	13	1
28	13	19	3	1	2	1	2	2	4	1
29	12	19	3	1	8	2	2	2	33	1
30	5	19	1	2	19	1	1	2	4	1
31	2	19	1	1	9	1	2	2	20	1
32	13	19	1	2	28	2	2	2	26	1
33	12	19	3	1	8	1	2	2	33	1
34	5	17	1	2	28	1	1	2	21	1
35	3	19	3	1	8	1	2	2	4	1
36	12	19	3	1	9	1	2	2	4	1

37	13	19	3	1	9	1	2	2	4	1
38	3	2	1	1	8	1	2	2	3	1
39	6	19	3	1	9	1	2	2	4	1
40	5	19	1	1	28	1	2	2	25	1
41	18	19	3	1	28	1	2	2	10	1
42	14	10	1	1	12	1	2	2	28	1
43	13	6	1	1	9	1	1	2	20	1
44	6	9	3	1	10	1	1	3	27	1
45	6	19	1	1	28	1	2	2	17	1
46	13	6	3	1	5	3	2	6	13	1
47	19	19	1	1	28	1	2	1	14	2
48	6	19	1	1	28	1	2	5	10	1
49	17	19	1	1	28	1	2	3	26	1
50	19	19	3	1	28	1	1	1	2	4
51	12	19	1	1	8	2	1	2	4	1
52	16	11	3	1	14	1	2	4	32	1
53	16	12	1	1	28	1	2	2	27	1
54	12	19	3	1	9	3	2	4	4	1
55	6	19	1	1	10	1	1	2	21	1
56	17	19	1	2	10	1	1	2	30	1
57	1	19	1	2	28	2	2	6	15	1
58	17	13	3	1	8	1	2	2	8	1
59	12	19	1	1	28	3	2	6	30	1
60	19	19	1	1	28	1	2	3	33	1
61	19	19	2	1	9	1	2	2	4	1
62	4	3	1	2	10	1	2	6	5	1
63	6	9	1	1	11	1	2	2	17	4
64	17	19	3	1	28	1	2	6	2	4
65	5	19	1	2	3	3	2	2	23	1
66	6	9	3	1	10	1	2	3	30	1
67	6	9	3	1	28	1	2	6	16	1
68	4	19	1	1	11	1	2	4	33	1
69	6	19	1	1	10	2	2	4	30	1
70	10	16	1	2	16	1	2	4	27	1
71	19	19	1	2	28	3	1	6	33	1
72	17	19	3	1	9	1	2	6	4	1
73	12	19	3	1	10	1	2	3	30	1
74	13	19	2	1	5	1	2	2	13	1
75	15	18	1	1	7	1	2	2	13	1
76	1	19	1	2	1	1	2	2	13	1
77	12	19	2	1	7	1	1	6	29	1
78	12	19	2	1	8	1	1	2	4	1
79	9	19	1	1	8	1	1	6	4	1

80	6	19	2	1	9	3	1	2	13	1
81	12	19	1	1	10	1	1	6	30	1
82	18	14	1	1	5	1	1	3	13	1
83	3	1	1	2	27	1	1	6	9	4
84	5	19	1	1	9	1	2	6	23	1
85	13	19	1	2	3	1	2	6	15	1
86	7	19	3	2	14	1	2	3	27	1
87	19	19	3	1	2	1	1	6	33	3
88	19	19	1	1	13	1	2	6	14	2
89	19	19	3	1	5	1	2	6	4	1
90	5	19	1	2	3	1	2	6	23	1
91	18	19	3	1	11	1	2	6	22	1
92	18	19	1	1	3	1	2	6	24	1
93	12	19	3	1	9	1	2	6	33	1
94	16	19	1	1	8	1	2	6	27	1
95	16	19	1	2	6	1	1	6	23	1
96	12	19	1	1	11	1	2	6	30	1
97	16	19	1	1	10	2	2	6	30	1
98	16	19	1	1	8	1	2	6	27	1
99	10	19	1	1	16	1	2	6	13	1
100	6	19	3	1	28	1	2	6	4	1
101	3	19	3	1	13	2	1	1	33	1
102	3	1	1	2	3	1	2	6	4	1
103	16	19	3	2	5	1	2	6	27	1
104	1	19	3	2	1	3	2	6	27	1
105	18	14	3	1	10	1	2	6	30	1
106	19	19	3	1	28	2	2	6	18	3
107	13	19	1	1	9	1	2	3	20	1
108	4	4	1	2	4	2	2	6	13	1
109	16	19	1	2	5	1	1	3	27	1
110	12	19	3	1	9	2	2	6	8	1
111	5	19	3	1	4	1	2	6	33	1
112	9	19	2	1	2	1	2	6	33	1
113	13	19	2	1	5	1	2	6	30	1
